# The acceptability of waiting times for elective general surgery and the appropriateness of prioritising patients

**DOI:** 10.1186/1472-6963-7-32

**Published:** 2007-02-28

**Authors:** Jurriaan P Oudhoff, Danielle RM Timmermans, Martin Rietberg, Dirk L Knol, Gerrit van der Wal

**Affiliations:** 1 Department of Public and Occupational Health, Institute for Research in Extramural Medicine, Free University Medical Centre, Amsterdam, The Netherlands; 2 Department of Primary Care & General Practice, University of Birmingham, Birmingham, UK; 3 Department of Surgery, Deventer Hospital, Deventer, The Netherlands; 4 Department of Clinical Epidemiology and Biostatistics, Free University Medical Centre, Amsterdam, The Netherlands

## Abstract

**Background:**

Problematic waiting lists in public health care threaten the equity and timeliness of care provision in several countries. This study assesses different stakeholders' views on the acceptability of waiting lists in health care, their preferences for priority care of patients, and their judgements on acceptable waiting times for surgical patients.

**Methods:**

A questionnaire survey was conducted among 257 former patients (82 with varicose veins, 86 with inguinal hernia, and 89 with gallstones), 101 surgeons, 95 occupational physicians, and 65 GPs. Judgements on acceptable waiting times were assessed using vignettes of patients with varicose veins, inguinal hernia, and gallstones.

**Results:**

Participants endorsed the prioritisation of patients based on clinical need, but not on ability to benefit. The groups had significantly different opinions (p < 0.05) on the use of non-clinical priority criteria and on the need for uniformity in the prioritisation process.

Acceptable waiting times ranged between 2 and 25 weeks depending on the type of disorder (p < 0.001) and the severity of physical and psychosocial problems of patients (p < 0.001). Judgements were similar between the survey groups (p = 0.3) but responses varied considerably within each group depending on the individual's attitude towards waiting lists in health care (p < 0.001).

**Conclusion:**

The explicit prioritisation of patients seems an accepted means for reducing the overall burden from waiting lists. The disagreement about appropriate prioritisation criteria and the need for uniformity, however, raises concern about equity when implementing prioritisation in daily practice.

Single factor waiting time thresholds seem insufficient for securing timely care provision in the presence of long waiting lists as they do not account for the different consequences of waiting between patients.

## Background

Over the last decades waiting lists for elective surgery have become a common feature of health care systems that are centrally organized and publicly funded. Although waiting lists can serve a purpose in rationing scarce resources, overly long delays of necessary treatment can have widespread negative consequences [1] and threaten the principles of providing timely and equitable access to care. Consequently, responsible health authorities have attached high importance to addressing problematic waiting times, amongst others in the United Kingdom, New Zealand, Canada, Sweden, and the Netherlands. Key initiatives that have been issued include the prioritisation of the patients on waiting lists [2-4] and the setting of maximal waiting time guarantees [5-10]. Despite the clear and shared purpose of these measures, it is unclear whether they adequately secure equity and timeliness in care provision, as empirical evidence to guide policy decisions in this area is often scarce or lacking.

The prioritisation of patients on the waiting list is intended to diminish the burden of waiting lists [11]. The far-reaching and diverse consequences of waiting lists, however, provide various medical, social, financial, and legal arguments to prioritise a patient. Several reports accordingly indicate that priority is assigned on the basis of a variety of clinical and non-clinical factors including personal preferences [12-19]. While uniform criteria for prioritisation are supported and adopted in some countries [4,5], there is still little evidence on the acceptance and equity of explicit prioritisation and on the ethical basis and the criteria that are considered appropriate for assigning priority.

Maximal waiting time guarantees for clinical treatment are a pragmatic means to secure timely care provision and to keep the consequences of waiting acceptable. The thresholds that are currently used, however, show a wide range and seem to be set rather arbitrarily. Whilst waiting time guarantees range between 3 and 6 months in the UK [5], Sweden [6], and New Zealand [8], the Dutch government, following a joint proposal of several medical organisations, set the maximum waiting time target for hospital treatment to 7 weeks, whereas 80% of the patients should be treated within 5 weeks [10]. These apparent differences in waiting time cut-off point, signify that most cut-off points are set fairly arbitrarily and it raises the question which waiting time thresholds would signify timely access to care and could from that viewpoint be deemed acceptable.

As the adequacy and acceptability of current waiting list policies is unclear, our study aims to outline the views and preferences of different stakeholders on various closely related aspects of prioritising patients and acceptable waiting times in care provision. We specifically studied the views of groups directly involved in care delivery and receipt in the area of elective surgery: patients, surgeons, occupational physicians (OPs), and general practitioners (GPs). Our study aims to assess their opinions on the following four aspects:

(1) the general acceptability of waiting lists for hospital care;

(2) the fairness of clinical prioritisation, and the appropriate ethical basis and methods for it;

(3) the acceptability of priority care based on non-clinical factors; and

(4) the maximally acceptable waiting times and their determinants for patients on elective surgical waiting lists.

Concerning the latter aspect our study focuses on waiting times for elective surgery for varicose veins, inguinal hernia, and gallstones. These three procedures were selected as they are known for lengthy waiting times within general surgery which comprises approximately 20% of the patients on hospital waiting lists [19-21].

While surgeons, OPs and GPs are all directly involved in the care delivery for surgical patients they clearly have different roles in it. Their specialties make them encounter slightly different patient populations on a daily basis. It can be expected these factors result in differences in their views on the appropriateness of criteria for prioritisation and on the acceptability of waiting times. With regard to prioritisation criteria it can therefore be hypothesised that the surgeons' primary role of restoring the patient's physical function will make them focus relatively more on these medical aspects. OPs, being responsible for the patient's return to work, may attach more importance to interferences with work when prioritising. For GPs, as generalists, and patients themselves a less distinct preference might be expected.

The patient populations seen by the different groups of doctors are likely to provide a frame of reference regarding judgements concerning the urgency of patients and the subsequent maximally acceptable waiting times. Accordingly, surgeons, who encounter proportionally more patients with more severe conditions than GPs and OPs, could find somewhat longer waiting times acceptable for the elective patient groups in the study. On a similar note, the patient groups, most directly affected by waiting, may be expected to be the least willing to find waiting acceptable.

## Methods

### Study sample

Postal questionnaires were sent out in May 2003 to the following groups:

- Three samples of 132 former patients who had been on a waiting list for surgical treatment of varicose veins, inguinal hernia, and gallstones respectively. These were randomly drawn from the participants in an earlier cross-sectional study on the consequences of waiting for surgery which started in mid 2001 and continued throughout 2002 [23]. The participants in that study were recruited from the waiting lists of 27 Dutch hospitals and appeared to be representative for the population of patients on hospital waiting lists for the 3 conditions of concern. Median waiting times these patients encountered were: 24 weeks for varicose vein surgery, 16 weeks for inguinal hernia surgery and 16 weeks for surgery for gallstones [23].

- A random sample of 200 surgeons (including trainees) derived from the registration of practising members of the Association of Surgeons of the Netherlands. Membership is held by the vast majority (> 90%) of surgeons practising in the Netherlands.

- Random samples of respectively 200 practising GPs, and 200 practising OPs derived from the registration and information division of the Royal Dutch Medical Association. These registrations contain all GPs and OPs practising in the Netherlands.

Sample sizes were determined on the basis of population size for the different groups of doctors (between 1000 and 2000) which were taken to require 80 to 100 persons per group to ensure a sufficient precision level for the purposes of our survey [24]. Based on previous experiences, response rates among patients were expected to be slightly higher.

### Study design and questionnaires

The questionnaire comprised two parts in addition to questions pertaining to sociodemographic details (e.g. age, sex, profession, and practice form (for doctors)). The first part of the questionnaire consisted of a list of statements that addressed ethical and practical aspects that emerged as important from literature and the public debate on waiting lists of the following topics: the general acceptability of waiting lists for hospital care (4 statements, see Results, Table 4); the fairness of clinical prioritisation (1 statement, see Results, Table 5) and the appropriate ethical basis (2 statements, see Results, Table 5) and methods for it (2 statements, see Results, Table 5); and the acceptability of priority care based on non-clinical factors (6 statements, see Results, Table 6). Participants were asked to indicate the degree to which they agreed with each statement on a 5-point scale: fully disagree/disagree to some extent/neutral/agree to some extent/fully agree. We computed mean scale scores from the participants' responses to assess their general attitude on two of the topics: the acceptability of waiting lists for hospital care (Cronbach's α = 0.74) and the acceptability of priority care based on non-clinical factors (Cronbach's α = 0.78). Deleting any of the items did not result in improvements of Cronbach's α of either scale. With 'neutral' coded as zero, the mean scale scores can range between -2 and 2.

The second part of the questionnaire addressed the participants' views on maximally acceptable waiting times for different patients on surgical waiting lists for varicose veins, inguinal hernia, and gallstones. For this part we constructed a series of different paper vignettes of patients for each disorder. These vignettes were designed according to conjoint analysis methodology which is used to analyse judgements on multi-attribute goods [25]. Each vignette described a patient by four characteristics: the physical symptoms, the psychological distress, the social limitations, and impairments in work. These characteristics were selected on the basis of interviews with experts on the consequences of waiting [1] and existing literature on generic priority criteria for waiting lists [16]. Three possible levels were defined for each characteristic, based on the range in outcomes of an earlier study on the consequences of waiting for surgery among patients with the three disorders [23]. The characteristics and their levels are shown in Table 1. Using a fractional factorial design (Orthoplan, SPSS 10.1) we selected the nine out of 81 (3^4^) possible vignettes per disorder that allowed regression analysis of the judgements on acceptable waiting times. Table 2 shows the composition of these nine vignettes. The participants had to consider each vignette independently and indicate their views on the maximum acceptable waiting time. A sample of the vignettes and the response categories as presented in the questionnaire is shown in Appendix A.

**Table 1:** The characteristics and their levels used to construct the vignettes of patients.

Characteristic		Level	Content
	*Varicose veins*	1	dislikes cosmetic appearance of varicose veins
		2	suffers occasionally from a feeling of heaviness in the leg
		3	suffers often from a feeling of heaviness and pain in the leg
Physical symptoms*	*Inguinal hernia*	1	a visible swelling during straining activities but no pain
		2	a nagging pain, occasionally
		3	continuous nagging sensations but also often severe pain
	*Gallstones*	1	less than 1 colic attack per month
		2	1 to 2 colic attacks per month
		3	2 colic attacks per week

Psychological distress		1	not worried
		2	somewhat worried about the symptoms and what might happen while waiting
		3	highly worried about the symptoms and what might happen while waiting

Social limitations		1	no limitations during usual social activities
		2	some limitations during usual social activities
		3	not able to perform usual social activities

Impairments in work		1	still able to work fully
		2	has to skip work partially (partially on sick leave)
		3	not able to perform job anymore

*Only the levels for the physical symptoms were different for each disorder.

**Table 2:** The composition of the nine vignettes by the levels of each characteristic*.

	Vign. #1	Vign. #2	Vign. #3	Vign. #4	Vign. #5	Vign. #6	Vign. #7	Vign. #8	Vign. #9
Physical symptoms	3	3	2	2	2	1	1	3	1
Psychological distress	2	3	1	3	2	3	1	1	2
Social limitations	3	1	3	2	1	3	1	2	2
Impairments in work	1	2	2	1	3	3	1	3	2

Vign. = Vignette

* The contents of the levels corresponding with numbers 1,2, and 3 are shown in Table 1)

### Procedure

To keep the number of appraisals manageable, each participant was presented with a randomly selected set of vignettes concerning two of the three studied disorders. Questionnaires for patients always included the vignettes on the disorder for which they had been on a waiting list. When patients appraised the vignettes concerning the other disorder, they were deemed to be laypersons. The order in which the disorders were presented in the questionnaire was varied randomly among participants. Variation was also applied to the order in which the vignettes of each disorder were presented.

The two parts of the questionnaire were presented to the participants as follows: firstly 9 vignettes of one disorder, then the list of statements, and finally the 9 vignettes of the second disorder.

Draft versions of the questionnaire were piloted among members of the general public and surgeons to test for completeness and comprehensibility.

The study was approved by the medical ethics committee of the VU University Medical Centre

### Analysis

To assess differences between the responses of groups to each statement and between the two mean scale scores we used Mann-Whitney tests and t-tests respectively. SPSS 10.1 was used for these analyses.

The judgements on acceptable waiting times for the vignettes of patients were analysed in two ways. Firstly, descriptive statistics were used to assess the minimum and maximum waiting times from the set of nine responses each participant provided per disorder. In the results section, these minimum and maximum responses are reported as the maximally acceptable waiting times for the vignettes to which the participant ascribed the highest urgency (shortest waiting time) and lowest urgency (longest waiting time) respectively.

Secondly, we performed multilevel ordered proportional odds regression analysis [26] of all responses to assess which factors influenced the participants' judgements on maximally acceptable waiting times for the vignettes (MLWiN 2.0 [27], estimation procedure: Markov chain Monte Carlo). For this analysis we recoded the responses into 6 categories: 2/5/7/10/15–20/and > 20 weeks. To address both the possible intra- and the interindividual variation in analysing the responses we used the following independent (explanatory) variables: the disorder and the levels of the 4 characteristics in the vignettes, the response group, and the participant's mean score for the scale reflecting the general attitude on the acceptability of waiting lists in health care. The latter variable was included in the analysis to see whether the judgements on a patient's urgency might go beyond a judgement of clinical severity and instead reflect the individuals' personal opinions towards the existence of waiting lists in themselves. This was deemed relevant given the highly elective nature of surgery for the conditions in our study. Possible effects arising from the study design (e.g. the order in which the disorders were presented) were corrected for if significant. Wald χ^2 ^tests were used to assess whether the effect of independent variables on the judgements on maximally acceptable waiting times was statistically significant (p < 0.05).

## Results

### Response

Altogether 518 of 996 (52%) mailed questionnaires were completed. The completed questionnaires of five doctors (1 surgeon, 2 OPs, and 2 GPs) were excluded as they were not practising anymore at the time of the study. Nine patients had also been on a waiting list for the disorder they were supposed to judge as a layperson. Their judgements concerning that disorder were excluded from the results. Final response rates and demographic details are shown in Table 3. The response rates among GPs and OPs raised some concerns about representativeness. Whilst anonymity of the respondents prevented from a formal non-response analysis among non responders, the representativeness of the participants was assessed by comparing their background variables with overall national statistics [28,29] which are given as a footnote to Table 3. The respondents' age largely matched those of the entire groups of GPs and OPs. For GPs response was relatively higher among women, whereas for OPs proportionally more men participated. In addition to these sociodemographics, the distribution of the type of practice among participating GPs matched the national distribution [28]: 27% worked in a single handed practice (vs. 27% nationally), 30.2% worked in a duo-practice (31% nationally) and the remainder worked in practices comprising > 2 GPs.

**Table 3:** Response numbers and demographic details of the participants.

		Response		Sex	Age	
		n	%	% male	Mean	(SD)
Patients	*Varicose veins*	82	64.6	29.3	54.0	(10.8)
	*Inguinal hernia*	86	65.6	94.0	64.4	(12.2)
	*Gallstones*	89	68.5	33.7	56.1	(13.1)
Surgeons (30.3% trainee)		100	50.0	91.0	43.5	(9.8)
OPs*		93	46.7	79.8	47.9	(6.8)
GPs**		63	31.5	64.6	46.5	(7.7)

Note: Eight patients of the original sample were left out when calculating the response rates as their questionnaires were returned undeliverable (moved house or deceased).

* National statistics for all OPs [29]: Age: 47.4 years (est. mean); Sex: 72.5% Male

** National statistics for all GPs [28]: Age: 47.8 years (est. mean); Sex: 70% Male

### The acceptability of waiting lists in health care (Table 4)

The positive mean scores on the scale indicate that most GPs and surgeons can accept the existence of waiting lists in health care. Oppositely, the patients responded on average slightly negatively to the statements on the acceptability of waiting lists in health care. Especially the statement that patients should not wait for care as they finance it found agreement by a majority of the patients. The mean scale score for the OPs did not differ significantly from zero (t_92 _= 1.23; p = 0.2) indicating on average a neutral attitude towards waiting lists. However, the relatively large standard deviation indicates that opinions were divided in this group. The distribution of the scores showed that 60% of the OPs had a positive scale score and 34% had negative scores.

**Table 4:** Grouped responses on the statements on the acceptability of waiting lists in health care (%, and mean scale scores).

		Patients n = 255	Surgeons n = 99	OPs n = 93	GPs n = 63
Waiting lists are an accepted part of an affordable health care service that is accessible for everyone.	Agree	50^ab^	59^a^	40^b^	59^a^
	Disagree	36	31	52	33
Since patients pay premiums for care, they should be given that care without having to wait.	Agree	64^a^	37^b^	47^c^	33^d^
	Disagree	21	51	37	60
Having to wait longer than two weeks for treatment is never acceptable.	Agree	43^a^	13^b^	22^c^	16^bc^
	Disagree	40	81	68	76
It is acceptable to have waiting lists even in a country as prosperous as the Netherlands.	Agree	44^a^	63^bc^	57^c^	71^b^
	Disagree	41	25	36	14
Scale scores for attitude towards the acceptability of waiting lists in health care	Mean* (SD)	-0.18^a ^(0.90)	0.50^b ^(0.97)	0.13^c ^(1.04)	0.65^b ^(0.81)

Note: Percentages do not add to 100 as response category "neutral" is not shown; the response categories "fully disagree" and "disagree to some extent" are combined into "disagree", and "agree to some extent" and "fully agree" are combined into "agree".

Note 2: The numbers of patients and surgeons do not correspond fully with overall response numbers. This difference is due to missing values on some statements (the number of missing values per statement never exceeded 3).

^a,b,c^: Non-corresponding superscripts between groups, indicate a significant difference in the responses (p < 0.05).

*Mean scale scores can range between -2 and 2 with zero indicating a neutral attitude; positive scores indicate a mean positive attitude.

### The fairness of clinical prioritisation and the appropriate ethical basis and methods for it (Table 5)

The majority in each group (51%–84%) found that prioritising patients on the waiting list would not be inequitable. Most participants (75%–94%) especially agreed to the prioritisation of patients based on the degree of suffering from symptoms. Less approval was found for the anticipated ability to benefit from treatment as a basis for prioritisation. However, a small majority of OPs (53%) agreed with this statement.

**Table 5:** Grouped responses (%) on the statements on the fairness of prioritisation, and the appropriate ethical basis and methods for it.

		Patients n = 255	Surgeons n = 99	OPs n = 93	GPs n = 63
Assigning priority to certain groups of patients on the waiting list is always unjustifiable.	Agree	34^a^	12^b^	12^b^	24^b^
	Disagree	51	84	79	71
If a patient has demonstrably more complaints as a result of an illness, he/she must be given priority.	Agree	82^a^	91^b^	75^a^	94^b^
	Disagree	11	5	11	0
A patient should be given priority if it is expected that he/she will benefit more from the treatment than another patient.	Agree	28^a^	41^a^	53^b^	33^a^
	Disagree	48	43	27	51
If certain patients are given priority, this can only be done in compliance with a nationally agreed system.	Agree	55^a^	30^b^	59^a^	62^a^
	Disagree	23	55	20	19
If it is allowed to prioritise patients, this works best if the physician can determine by him/herself which patients are given priority	Agree	43^a^	73^b^	42^a^	46^a^
	Disagree	41	16	37	33

Note: Percentages do not add to 100 as response category "neutral" is not shown; the response categories "fully disagree" and "disagree to some extent" are combined into "disagree", and "agree to some extent" and "fully agree" are combined into "agree".

Note 2: The numbers of patients and surgeons do not correspond fully with overall response numbers. This difference is due to missing values on some statements (the number of missing values per statement never exceeded 3).

^a,b^: Non-corresponding superscripts between groups, indicate a significant difference in the responses (p < 0.05).

The opinions about the possible methods of prioritisation showed that the surgeons had a different view on this than the other three groups. While most surgeons (73%) considered it best to leave the decisions on priority to the individual doctor, the patients (55%), the OPs (59%), and the GPs (62%) favoured a nationally agreed system for prioritising patients.

### Priority care on the basis of non-clinical factors (Table 6)

The mean scores on the scale for the acceptability of priority care based on non-clinical factors differed significantly between the response groups. A negative score was found particularly among patients (90%) and to a lesser extent among GPs (70%). The mean scores for the surgeons and OPs did not differ from zero (t_98 _= -0.75, p = 0.4; and t_92 _= 0.89, p = 0.4) indicating a neutral attitude. Among the surgeons, however, a majority (55%) agreed to assigning priority to hospital employees or personal acquaintances. The OPs particularly agreed with possibilities for priority care for employed patients.

**Table 6:** Grouped responses on the statements on the acceptability of priority care based on non-clinical factors (%, and mean scale scores).

		Patients n = 255	Surgeons n = 99	OPs n = 93	GPs n = 63
If a patient is given priority, this can only be done for medical reasons.	Agree	86^a^	42^b^	42^b^	67^c^
	Disagree	9	37	44	27
It must be possible to be operated earlier by paying extra (for example, in a private clinic)	Agree	28^a^	50^b^	62^b^	35^a^
	Disagree	63	35	27	51
Patients who occupy a high social position may be treated with priority.	Agree	7^a^	22^b^	19^b^	16^b^
	Disagree	89	64	58	74
A physician is allowed to give priority to personal friends and acquaintances or hospital staff on the waiting list.	Agree	6^a^	55^c^	19^b^	24^b^
	Disagree	89	27	52	60
Patients who are employed should be allowed to be given priority over patients who are not in paid employment.	Agree	29^a^	45^b^	65^c^	33^b^
	Disagree	62	41	26	44
An employer should be allowed to negotiate a financial agreement enabling an employee to be operated earlier.	Agree	27^a^	56^b^	82^c^	37^b^
	Disagree	57	34	12	46
Scale score for attitude towards priority care based on non-clinical factors	Mean* (SD)	-1.10^a ^(0.72)	-0.07^c ^(0.88)	0.07^c ^(0.78)	-0.55^b ^(0.82)

Note: Percentages do not add to 100 as response category "neutral" is not shown; the response categories "fully disagree" and "disagree to some extent" are combined into "disagree", and "agree to some extent" and "fully agree" are combined into "agree".

Note 2: The numbers of patients and surgeons do not correspond fully with overall response numbers. This difference is due to missing values on some statements (the number of missing values per statement never exceeded 3).

^a,b,c^: Non-corresponding superscripts between groups, indicate a significant difference in responses (p < 0.05).

*Mean scale scores can range between -2 and 2 with zero indicating a neutral attitude; positive scores indicate a mean positive attitude.

### Maximally acceptable waiting times

**Figure 1:**
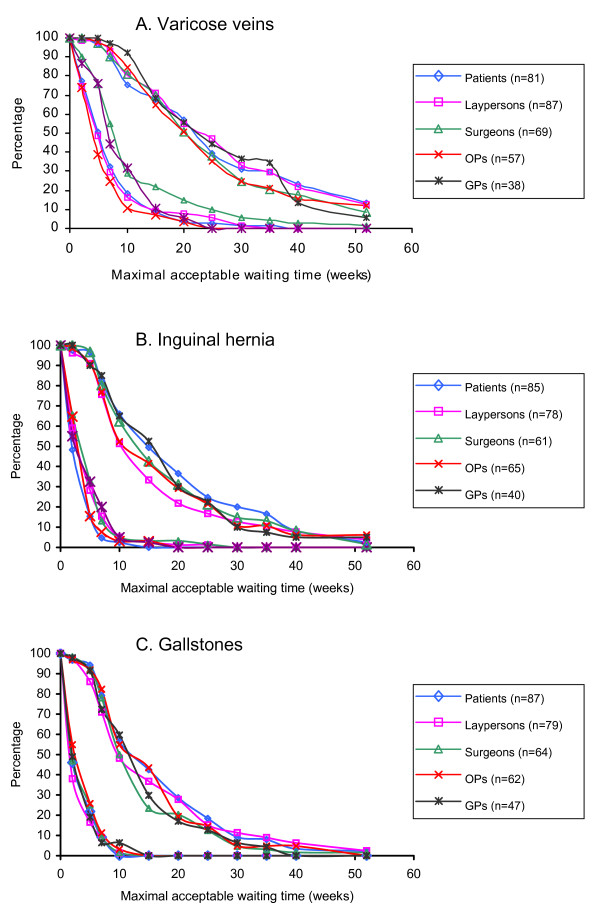
A-C. Distributions of the minimum and maximum responses in each group on acceptable waiting times for the nine vignettes of patients with varicose veins (a), inguinal hernia (b), and gallstones (c).

Figure 1, A to C show the distributions of the minimum and maximum of the responses that the participants of each group provided for the nine patient vignettes per disorder. For varicose veins, group means (medians) for the minimum responses on maximally acceptable waiting times ranged from 8.0 to 11.8 (5–10) weeks, whereas the mean maximum responses ranged from 22.1 to 26.3 (median 25) weeks. Similarly, for the vignettes for inguinal hernia the group means (medians) for the minimum responses ranged from 4.1 to 5.7 (2–5) weeks, and for the maximum responses from 15.8 to 20.1 (15–20) weeks. For gallstones, respective group means were 3.9 to 4.7 (2–5) weeks, and 14.4 to 16.9 (10–15) weeks.

The multilevel ordered proportional odds regression analysis showed that the waiting times that were judged maximally acceptable for the different vignettes were not related with the group which the participants belonged to (Table 7). The participants' individual score on the attitude scale on the acceptability of waiting lists in health care did have a significant effect on the responses. In concordance with the differences between the minimum and maximum responses, the participants' judgements on acceptable waiting times also depended significantly on the type of disorder in the vignettes, and the levels of the characteristics that described the vignettes of each disorder. While each characteristic affected the judgements on acceptable waiting times significantly, the largest impact was found for the degree of the physical symptoms and the impairments in work.

**Table 7:** Results from multilevel proportional odds regression analysis of the judgements on maximal waiting times for the vignettes of patients.

**Independent variables**	**Wald χ**^**2**^	OR*	**95%CI**
Response group	5.1 (df = 4, p = 0.281)		
*Patients (reference category)*		-	-
*Laypersons*		1.10	0.99 – 1.23
*Surgeons*		1.11	0.77 – 1.61
*Ops*		2.00	1.25 – 3.22
*GPs*		1.32	0.72 – 2.41
Disorder	1539.3 (df = 2, p < 0.001)		
Varicose veins (reference category)		*-*	*-*
*Inguinal hernia*		5.56	4.98 – 6.21
*Gallstones*		10.17	9.01 – 11.46
Physical symptoms	1275.9 (df = 2, p < 0.001)		
*Level = 1 (reference category)*		-	-
*Level = 2*		1.93	1.77 – 2.09
*Level = 3*		6.27	5.73 – 6.86
Psychological distress	114.9 (df = 2, p < 0.001)		
*Level = 1 (reference category)*		-	-
*Level = 2*		1.53	1.39 – 1.69
*Level = 3*		1.65	1.50 – 1.82
Social limitations	276.0 (df = 2, p < 0.001)		
*Level = 1 (reference category)*		-	-
*Level = 2*		1.54	1.40 – 1.70
*Level = 3*		2.31	2.08 – 2.56
Impairments in work	1532.8 (df = 2, p < 0.001)		
*Level = 1 (reference category)*		-	-
*Level = 2*		3.79	3.47 – 4.15
*Level = 3*		7.67	6.97 – 8.44
Attitude towards the acceptability of waiting lists in health care	34.4 (df = 1, p < 0.001)		
Scale score**		0.54	0.45 – 0.66
**Maximally acceptable waiting time**	Threshold		
*≤ 2 weeks*	*-7.71*		
*≤ 5 weeks*	*-5.59*		
*≤ 7 weeks*	*-4.18*		
*≤ 10 weeks*	*-2.65*		
*≤ 20 weeks*	*-0.53*		
*> 20 weeks (reference category)*	*-*		

*Cumulative odds ratios; odds ratios > 1 indicate a higher probability of assigning a response category involving shorter maximal acceptable waiting times for a vignette.

**A scale score of zero indicates a neutral attitude; positive scores indicate a positive attitude towards waiting lists in health care.

## Discussion

Long waiting lists for public health care services conflict with securing the principles of providing equitable and timely access to needed care. Most doctors in our study indicate that waiting lists could be an acceptable part of health care. The doctors especially agreed that waiting lists would not be irreconcilable with timely care. Responses on the other statements were more disparate. Patients seem on average less inclined to accept waiting lists in health care. On most statements the responses of patients show that opinions are divided about whether waiting lists are an acceptable entity in health care, indicating that on average waiting lists might certainly not be completely unacceptable. Only the statement that swift access to care should be provided because care is funded by their money did receive some more agreement among patients. This being an important argument against the acceptability of waiting lists clearly fits with the patients' consumer role in health care.

The prioritisation of patients on the waiting list is often advocated as a means to diminish the net burden from waiting lists [11]. In accordance with this, large majorities of each group in our study endorse the prioritisation of patients on the basis of clinical need. Conversely, only a small majority of OPs approve of prioritising patients based on ability to benefit, while patients, surgeons, and GPs show ambivalence or disapproval in this regard. This result contrasts with studies by Edwards et al. and Ridderstolpe et al. who found that the anticipated benefit should influence patient priority according to GPs, consultants, the general public, and health authority commissioners [30] and according to the opinions of physicians on priority setting in cardiac surgery [31]. These conflicting findings may be illustrative as the explicit use of ability to benefit for determining priority has also caused debate in New Zealand for it allegedly would contravene a just distribution of resources [16]. This controversy indicates that policy decisions on prioritisation require an explicit outline of the purpose and principles it should serve.

Regarding the practical implementation of prioritisation, patients, GPs, and OPs show a strong preference for explicitness and clarity on the process by favouring a system based on nationally agreed criteria. Surgeons on the other hand seem to prefer individual ownership over the process of prioritisation. This preference for individual responsibility may arise from concern about fallacies in the accuracy and sensitivity of systematic priority scoring tools in clinical practice which have been shown to exist [32,33]. Disapproval of imposed uniform criteria which have not proved to meet their purpose will likely provide a basis for gaming. It is therefore important that consensus need to be sought over which method for prioritisation is both deemed appropriate and can meet the desire for explicitness and transparency.

Preferential treatment for non-clinical reasons is controversial for it may conflict with fairness in care provision [19]. However, it has been shown to occur in practice and it can for instance contribute to reducing the costs of waiting times [34]. This ambiguousness may be reflected in our finding that surgeons and OPs show acceptance towards certain forms of preferential care, whereas patients and GPs generally disapprove of it. The found approval of preferential treatment among the surgeons and OPs is strongest for the aspects that either match their role or fall within a domain they control. This approval might thus either come from their expertise and experience or it might reflect the desire for individual ownership over prioritisation issues. Whereas the latter would likely entail arbitrariness in prioritisation, it seems necessary for health authorities to set clear guidelines on which non-clinical factors may and which may not be used for assigning priority.

In concordance with the approval of prioritisation based on clinical need, the waiting times that were deemed maximally acceptable differed widely depending on the disorder and the severity of the problems described in the patient vignettes. The average waiting times for the three disorders ranged from 2 to 7 weeks in case of severe symptoms and from 15 to 25 weeks for patients with few symptoms. Whereas other studies looking at the opinions of patients and doctors on acceptable waiting times for the patient groups in our study are lacking, studies on the patients' and doctors' views on maximum waits for hip and knee arthroplasty [35] and cataract surgery [36] show similar distinctions in the acceptable waiting times for patients with different symptom status. This diversity in acceptable waiting times obviously challenges the sufficiency of a single waiting time threshold to guarantee high quality health care.

Strikingly, the survey groups in our study had overall similar opinions on which waiting times would be maximally acceptable, regardless of their role in care delivery. This consensus indicates that a solid ground exists for defining timeliness of care between the different parties in the health care setting. Within each group, however, there was considerable variation in responses, especially when patient vignettes were deemed non-urgent. This inter-individual variation seems to be related to different opinions on the general acceptability of waiting lists in health care. Maximal waiting time guarantees may therefore need to reflect a gradient of appropriate waiting times which start at existing evidence on the clinical consequences of waiting and end at consensus over the timeliness of care according to local socio-medical culture.

### Limitations

In general, we consider the response numbers in our study to be acceptable, yet the response rate among GPs and OPs raises concern. The relatively long questionnaire and the fact that GPs and OPs will deal with surgical waiting lists less often than the surgeons themselves are factors that might have contributed to this lower response rate. The comparison with national statistics on basic demographics did not suggest that response was skewed in any particular direction. Also the diversity in opinions among GPs and OPs do not suggest that respondents represented a specific subgroup with a view on waiting lists. Yet it is unclear whether other differences between responders and non-responders were present. Some caution may thus be justified when drawing conclusions for these two groups.

The statements we used in our study reflected issues that emerged as important from the debate on waiting lists. Some statements may have addressed issues that apply to the Dutch health care setting but are less important elsewhere. Especially views on the acceptability of avoiding long waiting times in return for extra payment may be different in countries where private health care is common and such options hence already exist. In addition there is a possibility that some statements might have been open to differences in interpretation. Especially the question on prioritising acquaintances referred to both private friends and hospital personal. Although the principal behind this form of prioritisation might be the same, respondents may view these as distinctively different groups.

We used patient vignettes to be able to study the opinions on acceptable waiting times among a substantial number of persons of different backgrounds. We designed these vignettes on the basis of the outcomes of a study on the consequences of waiting among patients. Still, the vignettes are a simplified and abstract representation of real patients, which may have influenced the appraisals on acceptable waiting times.

## Conclusion

Patients, surgeons, OPs, and GPs support the prioritisation of patients based on clinical need and agree that it would not violate equity in care provision. Their views are, however, less univocal on the best method for determining priority and the acceptability of preferential treatment on non-clinical grounds. On both issues there seems to be a group who prefer individual responsibility and autonomy in care provision which can be accurate but may involve arbitrariness, while others desire uniformity and explicitness to secure equity in prioritisation. The implementation of prioritisation will therefore require either consensus on where these two diverse preferences can meet or decisions on which preference is deemed appropriate and valued highest.

Patients and doctors generally have similar opinions on which waiting times are maximally acceptable for elective surgical patients. Decisions on acceptable waiting times depend on the individual's acceptance of waiting lists in health care and the consequences of waiting for a patient. Timeliness in care provision may therefore need maximal waiting time guarantees that reflect socio-medical culture and account for the differences between patients.

## Competing interests

The author(s) declare that they have no competing interests.

## Authors' contributions

JO contributed to data collection, analysis and interpretation of the data and drafted the manuscript. DT contributed to study design, and analysis and interpretation of the results helped to draft the manuscript. MR contributed to study design, and the interpretation of the data helped to draft the manuscript. DK contributed to analysis and interpretation of data helped to draft the manuscript. GvdW contributed to the study design and the interpretation of the data helped to draft the manuscript. All authors read and approved the final manuscript.

## Appendix A

Example of a vignette and the response categories in the questionnaire. See Tables 8 and 9.

**Table 8:** Example of a vignette.

Miss Anchor, 48 years of age, consults the surgeon. She recently visited the GP for varicose veins.	Miss Anchor:
Miss Anchor **suffers occasionally from a feeling of heaviness in the leg **especially after standing for a while.	• **suffers occasionally from a feeling of heaviness in the leg**
She is **highly worried **that the symptoms will increase or that the varicose veins will enlarge during the wait and that surgery will not be effective than.	• **is highly worried about the symptoms and what might happen while waiting**
Miss Anchor encounters **some limitations during social activities**. She normally is a highly active member of a choral group, but she now has to cancel that regularly. She also visits her friends and family less frequently than normal.	• **has some limitations during usual social activities**
Miss Anchor is **still able to work fully**. The varicose veins do not interfere with her job in an employment agency.	• **is still able to work fully**

Below you find several possible waiting times.

Which waiting time do you consider maximally acceptable for miss Anchor?

**Table 9:** Waiting times questionnaire

Below you find several possible waiting times. Which waiting time do you consider maximally acceptable for miss Anchor?		
O unlimited waiting time	O 30 weeks	O 10 weeks
O 52 weeks	O 25 weeks	O 7 weeks
O 40 weeks	O 20 weeks	O 5 weeks
O 35 weeks	O 15 weeks	O 2 weeks

## Pre-publication history

The pre-publication history for this paper can be accessed here:


